# Correction to “Synthetic Biology‐Based Bacterial Extracellular Vesicles Displaying BMP‐2 and CXCR4 to Ameliorate Osteoporosis”

**DOI:** 10.1002/jev2.70095

**Published:** 2025-06-02

H. Liu, P. Song, H. Zhang, et al., “Synthetic Biology‐Based Bacterial Extracellular Vesicles Displaying BMP‐2 and CXCR4 to Ameliorate Osteoporosis,” *Journal of Extracellular Vesicles* 13 (2024): e12429, https://doi.org/10.1002/jev2.12429.

In Table 1 of the originally published article, the Characteristic of ECN, “Escherichia coli Nissle 1917,” is incorrect. This should read “*Escherichia coli* Nissle 1917 carrying the DE3 phage encoding for T7‐RNA Polymerase.”

In addition, Figure 2g in the originally published article is incorrect. The correct figure is shown below.


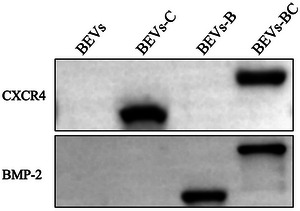


We apologize for this error.

